# Characterization of a putative *Plasmodium falciparum* SAC1 phosphoinositide-phosphatase homologue potentially required for survival during the asexual erythrocytic stages

**DOI:** 10.1038/s41598-017-12762-0

**Published:** 2017-10-05

**Authors:** Catherine Thériault, Dave Richard

**Affiliations:** Centre de recherche en infectiologie du CHU de Québec-Université Laval, Quebec City, Quebec Canada

## Abstract

Despite marked reductions in morbidity and mortality in the last ten years, malaria still takes a tremendous toll on human populations throughout tropical and sub-tropical regions of the world. The absence of an effective vaccine and resistance to most antimalarial drugs available demonstrate the urgent need for new intervention strategies. Phosphoinositides are a class of lipids with critical roles in numerous processes and their specific subcellular distribution, generated through the action of kinases and phosphatases, define organelle identity in a wide range of eukaryotic cells. Recent studies have highlighted important functions of phosphoinositide kinases in several parts of the *Plasmodium* lifecycle such as hemoglobin endocytosis and cytokinesis during the erythrocytic stage however, nothing is known with regards to the parasite’s putative phosphoinositide phosphatases. We present the identification and initial characterization of a putative homologue of the SAC1 phosphoinositide phosphatase family. Our results show that the protein is expressed throughout the asexual blood stages and that it localises to the endoplasmic reticulum and potentially to the Golgi apparatus. Furthermore, conditional knockdown and knockout studies suggest that a minimal amount of the protein are likely required for survival during the erythrocytic cycle.

## Introduction

Malaria is one of the most important infectious diseases worldwide with nearly half of the world’s population being at risk of contracting it^1^. In 2015, an estimated 212 million cases and 429 000 deaths were reported, much of these attributed to *Plasmodium falciparum*, the parasite responsible for the most serious form of the disease^2^. The rise of resistance to artemisinin-based combination therapies (ACT)^3^, combined with the absence of an approved efficient preventive vaccine, show the urge of finding new therapeutic targets. The symptoms of malaria are caused by the asexual red blood cell (RBC) stage of *P. falciparum*. The developmental processes occurring throughout the erythrocytic cycle involve extensive remodelling of both the parasite and the host RBC. Indeed, at the core of processes such as hemoglobin endocytocis, apicoplast and apical complex biogenesis, generation of new permeation pathways and export of the cytoadhesion complex, to name a few, is the parasite’s secretory pathway and substantial efforts have been put towards deciphering the mechanisms driving these processes^4,5^.

Despite being only minor components of cellular membranes, phosphoinositides (PIPs) play critical roles in numerous processes such as signal transduction, cell motility, cytoskeletal reorganisation, DNA synthesis, cell cycle regulation, adhesion, membrane transport, permeability and trafficking^6,7^. These lipids are found in seven isoforms depending on which positions on the inositol ring are phosphorylated through the action of kinases and phosphatases (reviewed in^8^). Though not much is known about PIPs and their effectors in *P. falciparum*, a few recent studies have demonstrated that they play critical roles in the development and survival of the parasite. For instance, phosphatidylinositol-3-phosphate (PI3P) and its kinase (*Pf*PI3K) are involved in hemoglobin endocytosis^9^, in apicoplast biogenesis^10–12^ and in artemisinin resistance^13^. Furthermore, PfPI4K is critical for membrane biogenesis and ingression during merozoite development^14^. In contrast to PIP kinases, nothing is known with regards to the parasite’s four putative PIP phosphatases identified through bioinformatics analysis^15^. Among these putative PIP phosphatases is a homolog of SAC1 (PF3D7_1354200), an enzyme cycling between the endoplasmic reticulum and the Golgi apparatus in yeast and mammalian cells with activity against PIP monophosphates^16,17^. In yeast, the absence of SAC1 is not lethal though it leads to membrane trafficking and actin dynamics defects^18^. In mammalian cells, SAC1 is an essential protein and a decrease in its expression causes Golgi and mitotic spindle disorganisation^19^. Knowing the important roles of SAC1 in other organisms, we decided to investigate the importance of the *P. falciparum* homologue in the erythrocytic cycle. Our results show that PfSAC1 is expressed throughout the asexual blood stages, that it localises to the endoplasmic reticulum and is enriched at or close to the Golgi apparatus. Interestingly, despite the fact that strongly decreasing PfSAC1 expression has no effect on parasite growth, our inability to inactivate the *PfSAC1* gene by regular knock out suggests that minimal amounts of the enzyme are essential for *in vitro* survival.

## Results and Discussion

### Identification of a *Plasmodium falciparum* putative SAC1 phosphoinositide-phosphatase homologue

**Figure 1:**
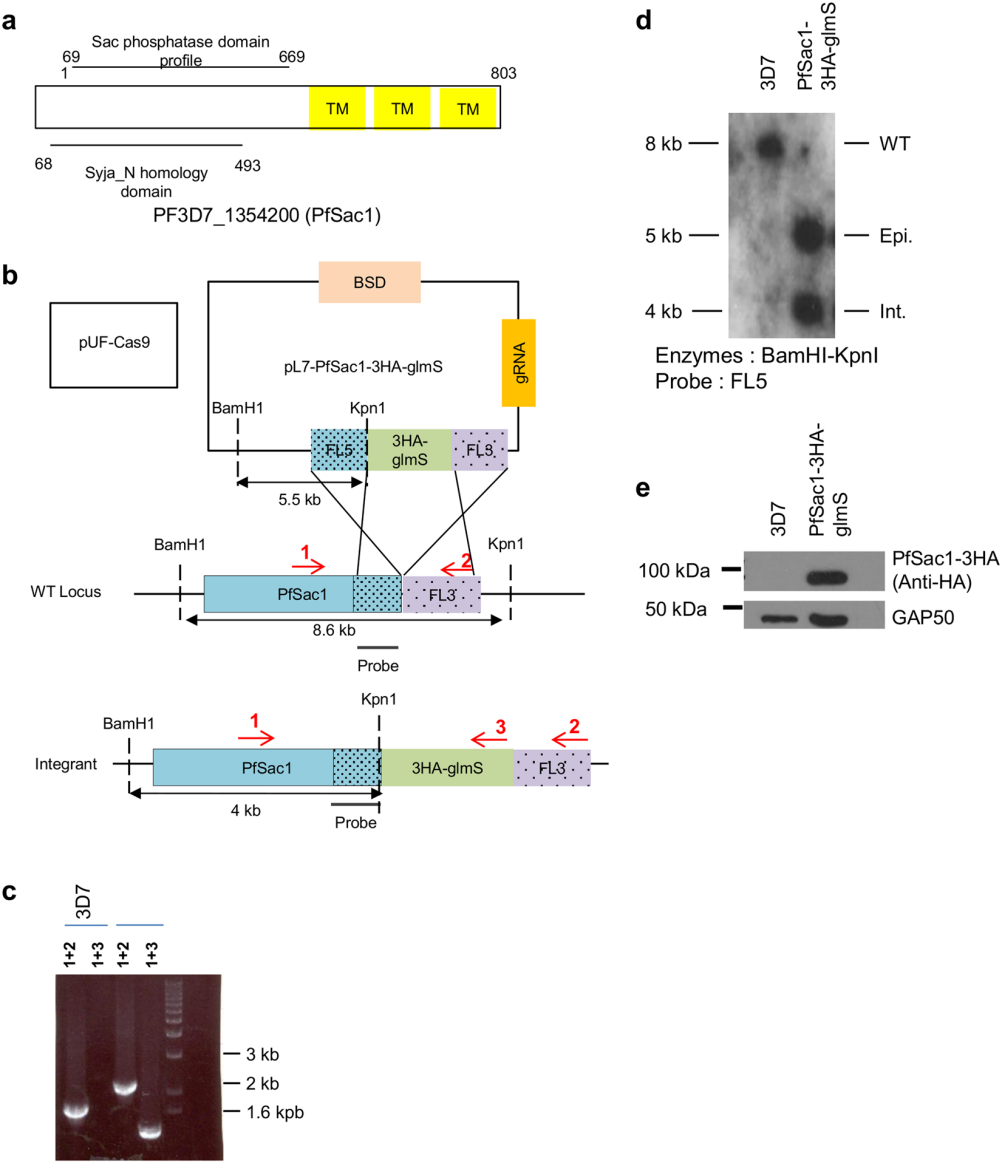
Generation of a PfSAC1-3HA-glmS tagged parasite line. (**a**) Schematic of the domain organisation of PF3D7_1354200 (PfSAC1) according to the PlasmoDB database. TM: Transmembrane domain. (**b**) Strategy, based on the CRISPR-Cas9 system, used to integrate the 3HA-glmS system at the 3′ end of the endogenous PfSAC1 gene. The expected sizes of digested products for Southern blot are shown. BSD: Blasticidin resistance gene. (**c**) PCR showing that our strain has integrated the cassette and that no WT gene is remaining. Primers used for amplification are shown on part b of the figure. Primer pair number 1 + 2 is for the detection of the wild type allele of the gene. Primer pair number 1 + 3 is to detect integration of the 3HA-glmS cassette at the 3′end of the *PfSAC1* gene. Primer 1: 5′upstream-gDNA-PfSAC1. Primer 2: 3′Spe1-PfSAC1-3′UTR. Primer 3: 3′glmS. (**d**) Southern blot showing the integration of the 3HA-glmS cassette at the 3′end of the PfSAC1 gene. The episomal from is still present, but no WT gene is remaining in the PfSAC1-3HA-glmS strain. Epi.: episome. Int.: integrant. (**e**) Western blot showing that PfSAC1-3HA is expressed in the integrant strain. Anti-HA and anti-GAP50 (as a loading control) antibodies were used. The uncropped blot is shown in Supplementary Figure 2a.

To identify putative *P. falciparum* homologues of the *S. cerevisiae* SAC1 phosphoinositide-phosphatase, a BLAST analysis was performed in the PlasmoDB database (www.plasmodb.org) using the full-length protein sequence. Three proteins with homology to ScSAC1 were recovered (PF3D7_1354200: score 6e-49, PF3D7_0802500: score 4e-30, PF3D7_0705500: score 1e-11), all of which contained the conserved CX_5_R(T/S) motif essential for catalytic activity^16^. ScSAC1 is a type II transmembrane protein with both N- and C-terminal domains facing the cytosol^20^. Of the three putative *P. falciparum* homologues, PF3D7_1354200 and PF3D7_0802500 have putative TM domains but only the former has them at the C-terminus, like ScSAC1 (Fig. 1a). In addition, proteomics analyses have shown that only PF3D7_1354200 was detectable in the blood stages^21–23^. For these reasons, we decided to name PF3D7_1354200 PfSAC1 and this protein is the focus of the present study.

### PfSAC1 is constitutively expressed throughout the asexual blood stages

To explore the role of PfSAC1 in the erythrocytic cycle, we used the CRISPR-Cas9^24^ system to tag the endogenous gene with a triple HA epitope combined with the glmS ribozyme to allow conditional regulation of expression^25^ (Fig. 1b). After positive selection with BSD, clones expressing PfSAC1-3HA were isolated and used for further characterisation. To determine whether proper integration of the 3HA-glmS cassette at the PfSAC1 locus had occurred, PCR amplification was performed with a primer binding upstream of the 5′ flank (FL5) (primer 1, see Fig. 1b) and a reverse primer specific for the 3HA-glmS cassette (primer 3, see Fig. 1b). As expected, no amplification was obtained in the 3D7 WT line while a band of approximately 1.5 kb was amplified in the PfSAC1-3HA-glmS strain (Fig. 1c), demonstrating that the PfSAC1 locus had been successfully tagged. To check whether untagged WT alleles were still present in the cloned PfSAC1-3HA1-glmS line, PCR amplification was done using primer 1 and a primer binding in the 3′flank (FL3) (primer 2, see Fig. 1b). A single band of 1.6 kb was obtained for the 3D7 WT strain whilst a band of 2 kb was obtained for the PfSAC1-3HA-glmS strain, in line with the addition of the 400 bp 3HA-glmS cassette at the PfSAC1 locus (Fig. 1c) We can therefore conclude that the 3HA-glmS cassette has been properly integrated at the 3′ end of the endogenous *PfSAC1* gene and that no WT version is remaining. This was also confirmed by Southern blot using a probe corresponding to FL5. As shown in Fig. 1d, we can detect the presence of the integration band at 4 kb, and an episomal band at 5.5 kb, despite the fact that the parasites have been off the selection drug for several weeks. An 8.2 kb WT band is only seen in the 3D7 WT strain, confirming the absence of a WT allele in our PfSAC1-3HA-glmS parasite line. To look whether the tagged protein was properly expressed, a Western blot with an anti-HA antibody was performed on total protein extracts of saponin-lysed mixed stage parasites and this revealed a single band at 95 kDa (Fig. 1e), which is around the expected size of 100 kDa for PfSAC1 fused with 3HA. These results demonstrate that we have succeeded in generating a parasite line where the WT allele of PfSAC1 has been tagged with 3HA and placed under the control of the glmS riboswitch.

**Figure 2:**
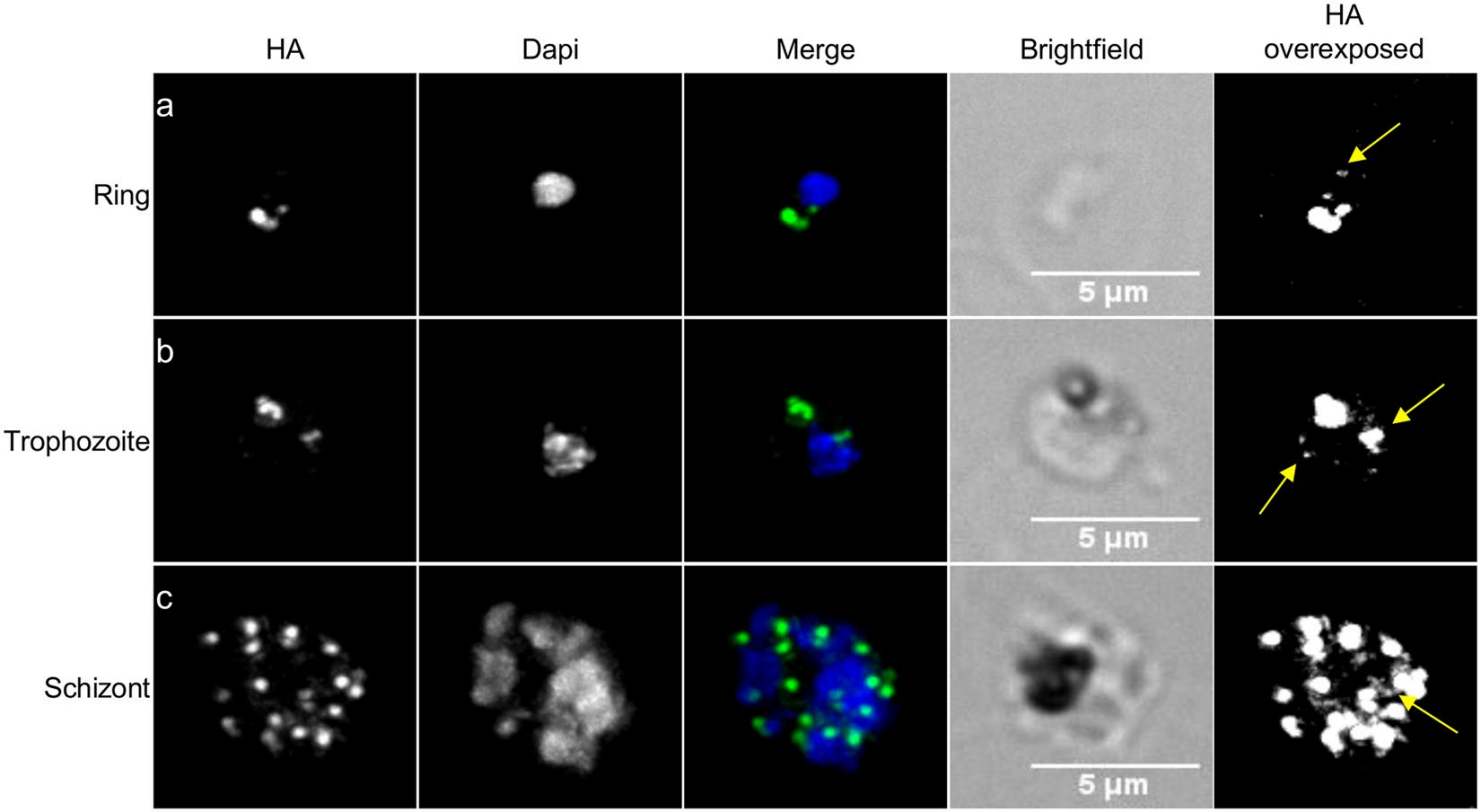
PfSAC1-3HA is constitutively expressed throughout the asexual erythrocytic life cycle. Immunofluorescence assay using anti-HA antibody showing that PfSAC1-3HA is expressed in ring (**a**), trophozoite (**b**) and schizont (**c**) stages. Merge shows the HA signal in green and the Dapi stained nucleus in blue. The HA overexposed panels are to highlight the additional faint labelling around the nucleus (yellow arrows). Scale bar is 5 μm.

To determine the expression pattern of the protein throughout the asexual blood stages, we performed immunofluorescence assays (IFA) using an anti-HA antibody (Fig. 2). As seen in Fig. 2a, in ring stage parasites, a bright spot of fluorescence is seen which increases as the parasite reaches the trophozoite stage (Fig. 2b). In schizonts, a high number of foci are now present (Fig. 2c). For all stages, some very faint signal surrounding some of the Dapi stained nuclei could be observed, reminiscent of the endoplasmic reticulum (ER)^26^ (Fig. 2, HA overexposed panels, yellow arrows). These results demonstrate that PfSAC1 is expressed throughout the asexual stages of the erythrocytic cycle, and that it is potentially found enriched at some regions of the ER.

### PfSAC1 is found in the endoplasmic reticulum and enriched at/close to the Golgi apparatus

**Figure 3:**
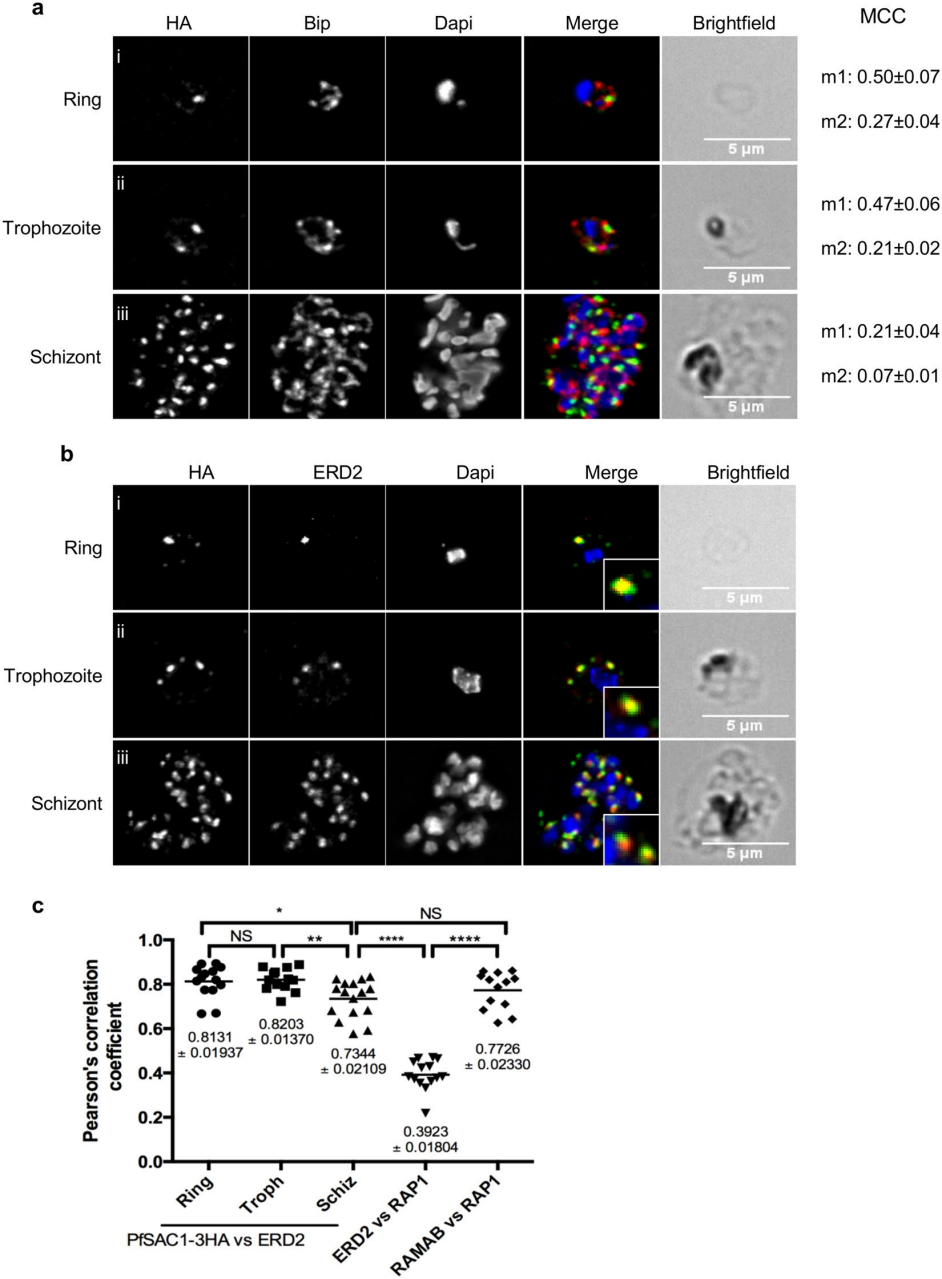
PfSAC1-3HA is found in the endoplasmic reticulum and enriched at or close to the Golgi apparatus. (**a**) Colocalisation analysis between PfSAC1-3HA and the endoplasmic reticulum marker Bip. Some overlap is observed between the two signals throughout the blood stages though it decreases in schizonts. MCC: Manders’ correlation coefficient. m1: HA/Bip. m2: Bip/HA. The mean ± SEM is represented for each condition, with n = 10. (**b**) Colocalisation analysis between PfSAC1-3HA and the Golgi apparatus marker ERD2. PfSAC1-3HA shows extensive overlap with ERD2 in rings (i) and trophozoites (ii) but this decreases in schizonts (iii). (**c**) Quantification of the colocalisation between PfSAC1-3HA and ERD2 at each stage. ERD2-RAP1 is used as a control for proteins residing in different organelles whilst RAMAB and RAP1 as a control for proteins from the same organelle. The coefficients were calculated by the intensity correlation of Alexa fluor 488 and 594. Each dot on the graph represents an individual cell. Horizontal line represents the mean. The mean ± SEM is represented for each condition. PfSAC1-3HA–ERD2: n_ring_ = 14, n_troph_ = 13, n_schiz_ = 16. ERD2 – RAP1: n = 14. RAMAB – RAP1: n = 13. NS: non significant, p-value = 0,1201. ****p-value < 0,0001. A two-way ANOVA and Tukey’s multiple comparisons tests were used to calculate the P-values.

To refine the subcellular localisation of PfSAC1-3HA, we performed IFAs with an anti-HA antibody and antibodies labelling different organelles of the parasite. As shown in Fig. 3a, PfSAC1-3HA overlaps with some parts of the ER in rings, trophozoites and schizonts, sometimes in close apposition/overlapping to foci of the ER marker Bip, as seen with mammalian and yeast SAC1 homologues^18,27,28^. Quantification of the colocalisation by Mander’s correlation coefficient^29,30^ revealed that in rings, 50 ± 7% of PfSAC1 colocalised with Bip whilst 27 ± 4% of Bip colocalised with PfSAC1 (Fig. 3a). In trophozoites, 47 ± 6% of PfSAC1 colocalised with Bip whilst 21 ± 2% of Bip colocalised with PfSAC1. This overlap decreased substantially in schizonts with 21 ± 4% of PfSAC1 colocalised with Bip and only 7 ± 1% for Bip vs PfSAC1. The pattern of PfSAC1-HA during parasite development from rings to schizonts being reminiscent of the Golgi apparatus dynamics previously observed in *P. falciparum* ^31–33^, we next investigated whether the concentration of PfSAC1-3HA colocalised with ERD2, a marker of this organelle^34^. As seen in Fig. 3bi, in ring stage parasites, the PfSAC1-3HA signal overlaps extensively with the Golgi apparatus signal. When the parasite goes from the trophozoite (3bii) to the schizont stage (3biii), the overlap between the two signals seems to decrease. To analyse this in a quantitative manner, we performed Pearson’s correlation analysis using at least ten different cells per condition. As a positive control for proteins residing in the same organelle, we performed colocalisation analysis with antibodies against RAMA and RAP1, two proteins of the bulb of the rhoptries, and obtained a r coefficient of 0.77. This value is explained by the fact that RAMA is found on the inside of the rhoptry bulb membrane while RAP1 is in the bulb^35,36^ so their respective signals never fully overlap. As a negative control, we used antibodies to proteins from two different organelles, ERD2 for the Golgi and RAP1 for the rhoptries, and obtained a coefficient of 0.39 (Fig. 3c and Supplementary Fig. 1). Analysis of the colocalisation between PfSAC1-3HA versus ERD2 shows r coefficients of 0.81 ± 0.02 in rings and 0.82 ± 0.01 in trophozoites, as high or even higher than the RAMA-RAP1 positive control. In schizonts, the level of colocalisation between the different cells analysed was quite more variable (from 0.58 to 0.83 with a mean of 0.73 ± 0.02). It was previously reported that, as the parasite matures from ring to schizont, there is a spatial segregation of the transitional ER, the cis-face and the trans-face of the Golgi^33^. Our results suggest that the PfSAC1 enriched focus might therefore potentially correspond to the tER. It is interesting to note that yeast and mammalian SAC1 shuttle between the ER and Golgi in a cell-growth dependent manner with the enzyme concentrating at the ER in dividing cells and translocating to the Golgi in resting cells or under starvation conditions^37–39^. To determine the substrate specificity of PfSAC1, we tried to express the phosphatase domain recombinantly but never managed to obtain the protein in a soluble form (results not shown). We additionally tried to immunoprecipitate the endogenous protein to perform phosphatase assays but could not solubilise it without the use of harsh detergents (results not shown). The ER localisation of ScSAC1 requires interaction between the ER enzyme dolicholphosphate-mannose synthase DPM1 and a hydrophobic motif in the C-terminus of SAC1^39^. *P. falciparum* possesses a DPM1 homologue however it is not functionally equivalent to *S. cerevisiae*’s and does not contain the C-terminal TM domain required for interaction with SAC1^40,41^. The presence of hSAC1 at the ER necessitates an interaction between a *KXKXX* motif at its extreme C-terminus and the COP1 complex^42^. Inspection of the PfSAC1 sequence shows the presence of a *KIKFI* motif directly upstream of the first predicted transmembrane domain but whether PfSAC1 interacts with COP1 remains to be tested. In conclusion, the strong overlap between PfSAC1 with the Golgi apparatus in rings and trophozoites and their increased segregation in schizonts suggests that the enzyme potentially associates with the transitional ER however a more detailed analysis using super resolution and immunoelectron microscopy would definitely be required to confirm or infirm this hypothesis.

### Minimal amounts of PfSAC1 are potentially required for survival of the asexual blood stages

**Figure 4:**
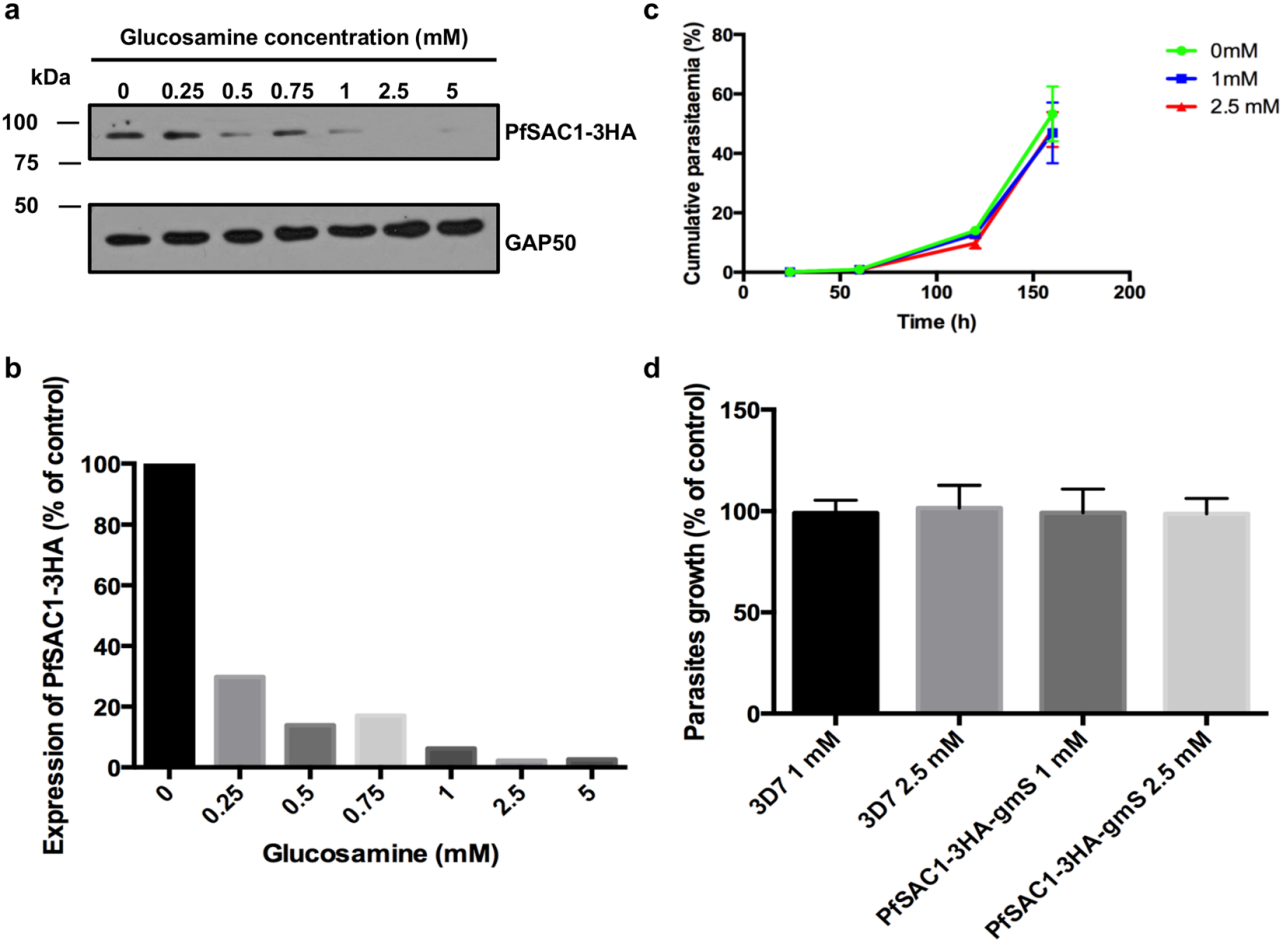
A minimal amount of PfSAC1-3HA is potentially required for asexual stage parasite survival. (**a**) Western blot showing that addition of glucosamine to the culture medium results in a dose-dependent knockdown of PfSAC1-3HA. GAP50 is used as a loading control. The uncropped blot is shown in supplementary Figure 2b. (**b**) Quantification of the extent of the PfSAC1-3HA knockdown by comparison to the loading control GAP50. (**c**) Knockdown of PfSAC1 does not have any effect on parasite growth. Parasitaemia was counted by FACS (fluorescence-activated cell-sorting) analysis on multiple cycles and under different glucosamine concentrations. Results from one experiment, representative of 3 independent experiments are shown. (**d)** Confirmation that glucosamine concentrations of 2.5 mM or less do not have a deleterious effect on either the PfSAC1-3HA-glmS line and the 3D7 control. The growth percentages were calculated with the T = 160 h data. The experiment was repeated three times with three triplicates by glucosamine concentration. We calculated the means of the triplicates and normalised the value with the 0 mM control. The growth percentage graph represents the mean of the ratio values obtained for all the experiments. Mean ± SEM for each condition.

To determine whether PfSAC1 plays an essential role in the asexual stages of the erythrocytic cycle, we made use of the glmS ribozyme conditional expression system^25^. When incubating tightly synchronous parasites with increasing concentrations of glucosamine (GlcN) from the ring to the schizont stage, a dose dependent decrease in the intensity of the HA signal is observed by Western blot (Fig. 4a). Densitometric analysis revealed that at 1 mM GlcN, a decrease of more than 95% of the PfSAC1-3HA signal was obtained and that at 2.5 mM, the HA signal was not detectable anymore (Fig. 4b). To assess if the decrease of expression of PfSAC1-3HA had an effect on parasite growth, we performed growth curve analyses (Fig. 4c). Despite PfSAC1-3HA levels being undetectable at 2.5 mM, there was no significant changes in the parasite growth in the presence of GlcN, like what was seen with the 3D7 control, (Fig. 4c and d). A variety of assays were next performed to characterise the knockdown strain in more detail and try to get some insight into the biological function of PfSAC1. By IFA, no morphological defects were seen when looking at the ER, Golgi, apicoplast and apical complex organelles and no decrease in the export of the virulence factor Erythrocyte Membrane Protein 1 was detected (results not shown). Finally, to investigate whether the expression of any of the other two putative PIP phosphatases was increased to compensate for the knockdown of PfSAC1, quantitative real-time PCR was performed but no difference was observed (results not shown). This suggests either that PfSAC1 is not essential or that residual undetectable levels are sufficient to perform its functions in the *in vitro* asexual erythrocytic cycle.

To determine whether PfSAC1 was completely dispensable for the erythrocytic cycle, we attempted a traditional knock out by single cross-over recombination using the newly developed selection-linked integration for targeted gene disruption strategy which greatly increases the efficiency of the selection of genomic integration^43^. The fact that we did not succeed in recovering parasites in six independent SLI-TGD experiments combined with our ability to tag the endogenous PfSAC1 locus with 3HA-glmS, demonstrating that it is amenable for genetic modification, suggests that a minimum amount of PfSAC1 is necessary and sufficient for proper *in vitro* survival of *P. falciparum* blood stage parasites. A conditional knock out strategy like the recently developed DiCre system^44,45^ would help to answer this question.

## Conclusion

In conclusion, our study identifies and provides an initial characterization of a putative SAC1 PIP-phosphatase in the malaria parasite *P. falciparum*. We show that the protein localises to the ER with an enrichment at or close to the Golgi apparatus, potentially representing the transitional ER. Furthermore, we show that a minimal amount of PfSAC1 is potentially essential for the asexual erythrocytic stage survival. A more definitive assessment of the function of PfSAC1 will require the use of conditional knock out systems.

## Materials and Methods

Study approved by the Canadian Blood Services (CBS) research ethics board, project number 2015.001 and by the CHU de Québec IRB, project number 2015–2230, B14-12-2230, SIRUL 104595. Written consent was obtained by the CBS for all study participants. All experiments were performed in accordance with relevant guidelines and regulations.

### Parasite Culture

*P. falciparum* 3D7 parasites were originally obtained from David Walliker at Edinburgh University. *P. falciparum* asexual stage parasites were cultured under standard conditions in RPMI-HEPES medium at 4% hematocrit (human erythrocytes of O^+^ group) and 0.5% (w/v) Albumax^TM^ (Invitrogen)[1]. They were kept at 37 °C in a gas mixture of 5.0% oxygen, 5.0% carbon dioxide and 90% nitrogen.

### Vector Construction, Transfection and Southern Blotting

To create the plasmid pL7-PfSAC1-3HA-glmS-BSD, the *hDHFR* gene of the pL6-eGFP plasmid^24^ was first replaced with the *BSD* gene to make pL6-BSD. The 3HA-glmS was synthesized with flanking restriction sites 5′Not1-Xho1-Xma1 and 3′Spe1-SacII and cloned in the pL6-BSD plasmid digested with Not1-Spe1, replacing the original FCU expression cassette. The FL5 fragment of PfSAC1 was amplified with primers 5′Xho1-1406-PfSAC1 and 3′Xma1-mutstopless-PfSAC1 and cloned in frame with 3HA in pL6-3HA-glmS-BSD digested Xho1-Xma1. The FL3 fragment was amplified with primers 5′SacII-PfSAC1-3′utr and 3′Spe1-PfSAC1-3′utr and cloned in pL6-PfSAC1FL5-3HA-glmS-BSD digested with SacII-Spe1. To make the PfSAC1 gRNA, primers forgRNASAC1 and revgRNASAC1 were annealed as described previously^24^ and the resulting gRNA was cloned by InFusion in pL6-PfSAC1FL5-3HA-glmS-FL3-BSD digested with BtgZ1 to generate pL7PfSAC1-3HA-glmS-BSD.

Parasites were transfected and integrants were selected as described previously with some modifications^46^. Briefly, *P. falciparum* 3D7 parasites were transfected with 100 ug each of purified pL7PfSAC1-3HA-glmS-BSD and pUF-Cas9 plasmids (Promega). Positive selection for transfectants was achieved using 2.5 mg/ml BSD (Sigma-Aldrich) for 5 days after which the drug was removed. Integration was monitored by Southern blot according to standard procedures and by PCR using the forward 5′upstream-gDNA-PfSAC1 (primer 1) and the reverse 3′-glmS primers (primer 3). Primer 1 was used with the reverse 3′Spe1-680-PfSAC1-3′UTR primer (primer 2) to detect the WT version of the gene. (see Table 1 for primer sequences).

**Table 1:** List of primers used.

**Name**	**Sequence**
5′upstream-gDNA-PfSAC1	GCGTATATTAAATTTACATATG
3′-glmS	GGTACCAGATCATGTGATTTCTCTTTG
5′Xho1-1406-PfSAC1	CCCTCGAGGTGATTGTAATGTAGAAC
3′Xma1-mutstopless-PfSAC1	AACCCGGGAGAATTGGCGTCTAATTTTGGAGATGAAATGAC
5′SacII-PfSAC1-3′utr	TACCGCGGAAATGAAAAAATAAATAATATATATATATATAATT
3′Spe1-PfSAC1-3′UTR	GCGACTAGTCATATAACTATAAATGATCTATCCTTTC
ForgRNAPfSAC1	TAAGTATATAATATTAGTCATTTCATCTCCTAAATTGGGTTTTAGAGCTAGAA
RevgRNAPfSAC1	TTCTAGCTCTAAAACCCAATTTAGGAGATGAAATGACTAATATTATATACTTA
5′Not1-PfSAC1	CCGCGGCCGCTAAACGAATTACTCTAAAATTCCTTT
3′Mlu1-513-PfSAC1	CCACGCGTGTTGACTGAAATAAGTTATCAAAAT

-Restriction sites are underlined.

### Western Blotting

Parasites were synchronized twice at an 18-hour interval so that the remaining parasites in culture were at the late ring stage (between 18 and 22 hours), with a 0.3 M alanine-10mM HEPES solution (as described in^47^). Synchronous parasites were then harvested by saponin lysis, the pellets solubilised in SDS protein sample buffer and separated on a 7.5% SDS-PAGE gel under reducing conditions and transferred to PVDF membranes (Millipore). The antibodies (rabbit serum anti-GAP50 1:2000^48^, mouse monoclonal anti-HA, (Cedarlane, 1:2000, CLH104AP) were diluted in 0.1% (v/v) Tween 20-phosphate-buffered saline with 1% (w/v) skim milk. Appropriate HRP-coupled secondary antibodies were used and immunoblots were revealed by ECL (Amersham Biosciences). For all expression analyses, proteins extracted from an equal number of cells were used for each time point.

### Fluorescence Imaging

Fluorescence images of parasites were captured using a GE Applied Precision Deltavision Elite microscope with 100×1.4NA objective and with a sCMOS camera and deconvolved with the SoftWorx software. Pearson’s correlation coefficients were calculated with the Fiji software^49^ on regions of interests of image stacks, including zero-zero pixels and without thresholding. For the colocalisation analysis between PfSAC1 and the ER marker Bip, Mander’s correlation coefficients were calculated with the Fiji software on regions of interests of image stacks, with manual thresholding. Chromatic calibration of the microscope was performed prior to imaging experiments. For immunofluorescence assays, parasites were fixed on slides using 4% paraformaldehyde (ProSciTech)^50^. After blocking in 3% bovine serum albumin (Sigma Aldrich) the cells were incubated for 1 hour with rabbit polyclonal anti-ERD2 1:2000^34^, mouse monoclonal anti-HA 1:2000 (Cedarlane, HA.C5), or rabbit polyclonal anti-Bip 1:500 (Sabrina Absalon and Jeffrey Dvorin, unpublished). Bound antibodies were then visualised with Alexa Fluor-594 anti-rabbit IgG and Alexa Fluor-488 anti-mouse IgG diluted 1:1000. Parasites were mounted in Vectashield (Vecta Laboratories) containing with 0.1 μg/ml 4′, 6–diamidino-2-phenylindole (Dapi, Invitrogen). Images shown represent the maximum projection of all z stacks.

### Conditional Knockdown analysis

To perform the conditional knockdown of PfSAC1-3HA, tightly synchronous ring stage parasites were incubated with glucosamine (Sigma-Aldrich), at different concentrations (0 mM; 0.25 mM; 0.5 mM; 0.75 mM; 1 mM; 2.5 mM; 5 mM), until they reached the schizont stage at which point they were harvested by saponin lysis. The resulting pellets were solubilised in SDS sample-buffer to extract the proteins and analysed by Western blot. To investigate if the knockdown of PfSAC1-3HA had an effect on parasite growth, the same experiment was performed but the cultures were allowed to grow over multiple cycles. Tightly synchronous ring stage PfSAC1-3HA parasites were seeded at 0.5% parasitaemia and grown with multiple glucosamine concentrations (0 mM, 1 mM, 2.5 mM). After 24, 60, 120 and 160 hours in culture, the cells were harvested and analysed by fluorescence-activated cell sorting (FACS) on a BD FACSCanto A to calculate the parasitaemia. Briefly, the cells were stained with SYBRGold (Invitrogen-Molecular Probe) and then fixed with 1% paraformaldehyde for 1 hour. 100 000 events were acquired on the FACSCanto A using the FACSDiva software. The data was analyzed with the FlowJo software.

### Knock out attempts by single cross-over using selection linked-integration for targeted gene disruption (SLI-TGD)

A 500 bp homology region from the N-terminus of PfSAC1 was amplified from 3D7 genomic DNA with flanking restriction sites 5′Not1 and 3′ Mlu1. This fragment was then cloned in the pSLI-TGD vector. 3D7 WT parasites were transfected and selected with 5 nM WR99210. Once obtained, the transfectants containing episomal plasmids were diluted to 2% parasitaemia and put on 400 μg/ml G418 to select for parasites having integrated the construct. These cultures were then fed daily for 10 days and then every other day for 5 weeks after which they were discarded since no parasites had reappeared. Proper integration resulting in disruption of the targeted gene leads to the expression of GFP and the neomycin phosphotransferase marker so only integrants are able to grow on G418. Failure to recover parasites after G418 selection is therefore indicative of a potentially essential gene^43^.

## Electronic supplementary material

Supplementary figures
