# Supplementary material for: Characterization of a putative Plasmodium falciparum SAC1 phosphoinositide-phosphatase homologue potentially required for survival during the asexual erythrocytic stages
Source: Sci Rep. 2017 Oct 5;7:12710. doi: 10.1038/s41598-017-12762-0 (PMC5629215; doi:10.1038/s41598-017-12762-0)
Supplement: Supplementary file 1 — Supplementary figures [file 41598_2017_12762_MOESM1_ESM.pdf]

Supplementary information

Characterization of a putative *Plasmodium falciparum* SAC1 phosphoinositide-phosphatase homologue potentially required for survival during the asexual erythrocytic stages.

Catherine Thériault[1] and Dave Richard[1]*

[1] Centre de recherche en infectiologie, CHU-Université Laval

Quebec City, Quebec, Canada

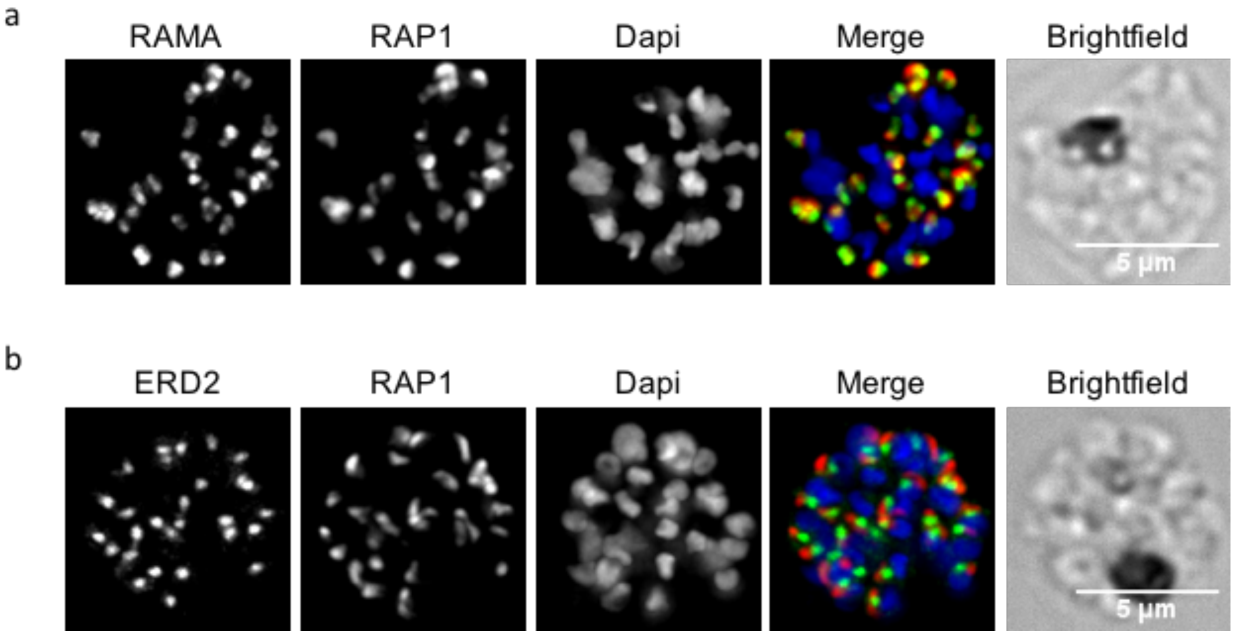

Supplementary Figure 1: A) Colocalisation analysis between the rhoptry membrane marker RAMA and the rhoptry bulb marker RAP1. The merge image shows RAMA in green and RAP1 in red. B) Colocalisation analysis between the Golgi marker ERD2 and the rhoptry bulb marker RAP1. The merge image shows ERD2 in green and RAP1 in red. Blue represents the Dapi stained nucleus.

a

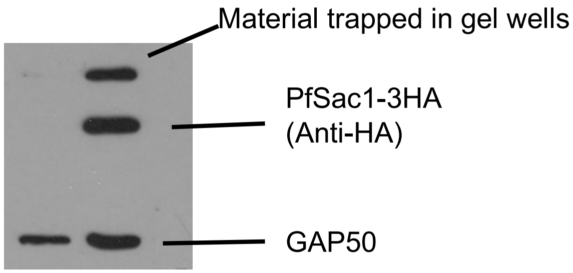

Uncropped Western blot related to figure 1e

b

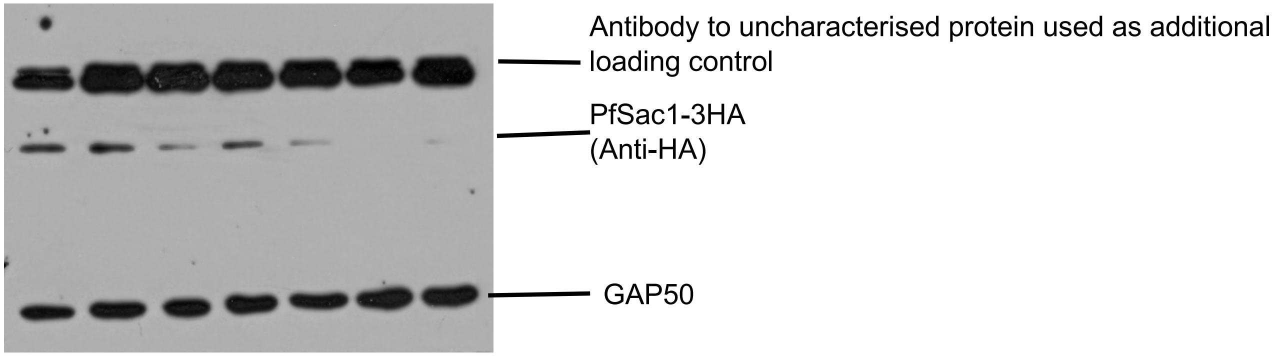

Uncropped Western blot related to figure 4a
